# Study of the Molecular Dynamics of Multiarm Star Polymers with a Poly(ethyleneimine) Core and Poly(lactide) Multiarms

**DOI:** 10.3390/ma10020127

**Published:** 2017-02-04

**Authors:** Frida Román, Pere Colomer, Yolanda Calventus, John M. Hutchinson

**Affiliations:** Laboratori de Termodinàmica i Físicoquímica, TERFIQ, Departament de Màquines i Motors Tèrmics, ESEIAAT, Universitat Politècnica de Catalunya, Barcelona Tech, Carrer Colom 11, Terrassa 08222, Spain; colomer@mmt.upc.edu (P.C.); calventus@mmt.upc.edu (Y.C.); hutchinson@mmt.upc.edu (J.M.H.)

**Keywords:** dielectric relaxation spectroscopy (DRS), multiarm star polymers, poly(lactide) (PLA), poly(ethyleneimine) (PEI), differential scanning calorimetry (DSC)

## Abstract

Multiarm star polymers, denoted PEI*x*-PLA*y* and containing a hyperbranched poly(ethyleneimine) (PEI) core of different molecular weights *x* and poly(lactide) (PLA) arms with *y* ratio of lactide repeat units to N links were used in this work. Samples were preconditioned to remove the moisture content and then characterized by thermogravimetric analysis (TGA), differential scanning calorimetry (DSC) and dielectric relaxation spectroscopy (DRS). The glass transition temperature, *T*_g_, is between 48 and 50 °C for all the PEI*x*-PLA*y* samples. The dielectric curves show four dipolar relaxations: γ, β, α, and α′ in order of increasing temperature. The temperatures at which these relaxations appear, together with their dependence on the frequency, allows relaxation maps to be drawn, from which the activation energies of the sub-*T*_g_ γ- and β-relaxations and the Vogel–Fulcher–Tammann parameters of the α-relaxation glass transition are obtained. The dependence of the characteristic features of these relaxations on the molecular weight of the PEI core and on the ratio of lactide repeat units to N links permits the assignation of molecular motions to each relaxation. The γ-relaxation is associated with local motions of the –OH groups of the poly(lactide) chains, the β-relaxation with motions of the main chain of poly(lactide), the α-relaxation with global motions of the complete assembly of PEI core and PLA arms, and the α′-relaxation is related to the normal mode relaxation due to fluctuations of the end-to-end vector in the PLA arms, without excluding the possibility that it could be a Maxwell–Wagner–Sillars type ionic peak because the material may have nano-regions of different conductivity.

## 1. Introduction

Cured epoxy resins are thermosets exhibiting a cross-linked structure with excellent electrical properties, good chemical and corrosion resistance, low shrinkage, and high tensile strength and modulus. On account of these characteristics, epoxy resins are widely used in applications such as coatings, adhesives, and encapsulation of electronic components. To obtain an acceptable set of properties, the oligomers or monomers of low molecular weight must be converted into a highly cross-linked network polymer; this is achieved by means of curing agents, which involve a variety of reaction mechanisms [1,2,3]. The wide range of curing agents available for their cross-linking makes the resulting cured epoxy systems extremely versatile, and in particular there is considerable interest in the use of dendrimers and hyperbranched polymers as cross-linking agents.

Dendritic polymers are materials with a highly branched structure. Due to the large number of end groups, one at the end of every branch, they are highly functional. The high degree of branching renders entanglement of the polymers impossible which results in low melt and solution viscosity. Dendritic architecture may be divided into six subclasses: dendrons and dendrimers, linear-dendritic hybrids, dendrigrafts or dendronised polymers, hyperbranched polymers, multi-arm star polymers, and hypergrafts or hypergrafted polymers. The first three subclasses exhibit perfect structures with a degree of branching of 1.0, while the latter three exhibit a random branched structure. 

Dendrimers are molecules with a three-dimensional globular shape [4,5,6] and belong to the family of highly branched molecules emanating from a central core with a large number of functional terminal groups at their periphery. Recently, dendrimers have been used as modifiers in thermoplastic polymers and as curing agents for thermosetting materials. For example, Tande et al [7] used ester-terminated poly(propyleneimine) dendrimers to plasticize polyvinyl chloride (PVC), while other authors report that poly(amidoamine) dendrimers, butyl-glycidylether-modified poly(propyleneimine) dendrimers and others of similar molecular structures could effectively cross-link epoxy resins [8,9,10,11,12,13].

Such star-like topologies have considerable interest because of their unusual physical and rheological properties. The properties of the final materials and their processability can be tailored as a function of the core and arms structures, the type and amount of functional end groups, the molecular weight, and the length and number of the polymeric arms. In previous studies, multi-arm stars with poly(ε-caprolactone) arms using hyperbranched poly(glycidol) or poly(ethyleneimine) as a core have been synthesized and studied as modifiers in the curing of diglycidylether of bisphenol A (DGEBA) epoxy resin using cationic and anionic initiators as curing agents [14,15,16].

Recently, hyperbranched poly(ethyleneimine) has been used as a multifunctional cross-linker of epoxy resins. The densely branched architecture and high molecular weight of poly(ethyleneimine) create significant mobility restrictions, which have a strong effect on the glass transition temperature and on the degree of cross-linking of the cured materials [17]. Within the last few years, multiarm stars with poly(lactide) (PLA) arms using a poly(ethyleneimine) (PEI) of different molecular weight (Lupasol^®^, BASF, Mississauga, ON, Canada) as the multifunctional core have been synthesized [18]. With an appropriate selection of PLA arms, which contain rigid esters of secondary alkyl groups in the structural units, materials with higher glass transition temperature can be obtained, compared with the use of similar stars with poly(ε-caprolactone) (PCL) arms with primary ester linkages. Such materials formed by a multifunctional core of PEI of different molecular weights and multiple branches of PLA, denoted PEI*x*-PLA*y* where *x* represents the molecular weight of the poly(ethyleneimine) and *y* represents the ratio of lactide repeat units to N links, were used as chemical modifiers in the anionic curing of DGEBA epoxy resins. The addition of the multiple arm stars led to homogeneous materials with an improvement of the impact strength in comparison with the neat material, without compromising the glass transition temperature [18].

**Figure 1:**
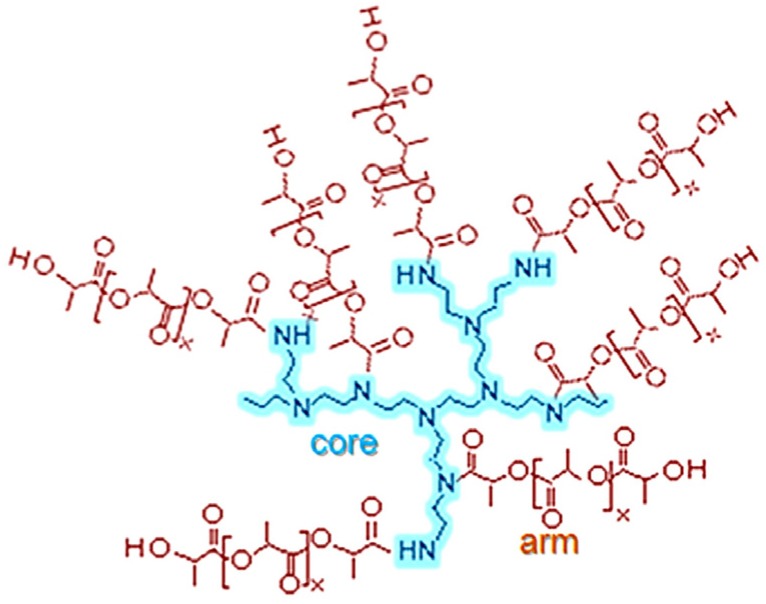
Scheme of multiarm star copolymers PEI*x*-PLA*y*.

In previous work, we studied the dielectric relaxations associated with pure PEI and blends of PEI and DGEBA epoxy resin in the uncured state, during the curing reaction, and as a fully cured system. The behavior of each relaxation was analyzed in order to ascertain the molecular motions and the groups responsible for these relaxations [19]. Likewise, the molecular mobility of poly(lactide) has also been studied, including for example the relationship between morphology and glass transition [20]. On the other hand, for the PEI*x*-PLA*y* copolymers, there has been no systematic study of the effect of, for example, the molecular weight of the PEI core, or of the effect of the presence of the branches of PLA and the length and density of these branches. Accordingly, in the present work the thermal and dielectric behavior of three multiarm star polymers composed of a polyethyleneimine (PEI) core and polylactide (PLA) arms has been studied, and has been compared with that of a commercial –NH_2_ terminated hyperbranched polymer, also based on polyethyleneimine but with different molecular weights, in order to identify the contributions of the core and arm architectures to the molecular dynamics. A schematic illustration of the molecular architecture of these star-shaped polymers is shown in Figure 1.

## 2. Results and Discussion

**Figure 2:**
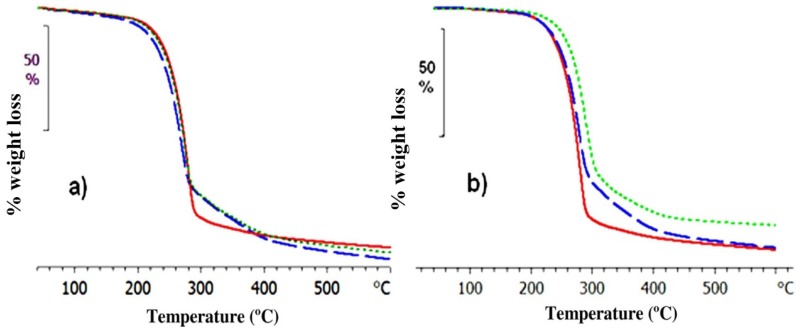
Thermogravimetric results, % weight loss as a function of temperature of the samples PEI2000-PLA20 (continuous line), PEI2000-PLA5 (dashed line) and PEI800-PLA5 (dotted line): (**a**) original samples; (**b**) isothermally preconditioned samples.

Figure 2a,b show the thermograms obtained by thermogravimetric analysis (TGA, Mettler-Toledo AG, Schwerzenbach, Switzerland), showing the thermal degradation behavior of the samples PEI2000-PLA5, PEI2000-PLA20, and PEI800-PLA5. Two kinds of experiments were performed in order to observe the effect of moisture in the samples: in the first type of experiment, the original samples were heated at 10 °C/min over the temperature range from 40 to 600 °C; in the second type of experiment, the original samples were first subjected to an isothermal pre-conditioning at 100 °C for 30 min, followed by cooling to 40 °C at 20 °C/min, before monitoring the TGA scan on heating from 40 to 600 °C at 10 °C/min.

Appreciable differences in the thermal degradation of the original samples and preconditioned samples can be seen in these Figures. In particular, there is a large difference in the temperature corresponding to 2% weight loss (*T*_D2%_) between the original and the preconditioned samples, as shown in Table 1, as a consequence of the moisture content in all the samples studied. A comparison of Figure 2a,b shows that this moisture content has more influence on the thermal stability of the PEI800-PLA5 and PEI2000-PLA5 polymers, which is also reflected in the values of *T*_onset_. In Figure 2b, it can be seen that the star polymer PEI800-PLA5, free of moisture, is thermally more stable in comparison with the other samples. The thermal degradation of these samples occurs in two stages. The first stage, which is the more pronounced and occurs around 260 °C, corresponds to the decomposition of the PLA arms. Previous studies have shown that PLA is a thermally degradable polymer as a consequence of the presence of ester groups of secondary alkyl moieties in the main chain, which can break by a thermal β-elimination process in the temperature interval from 200 to 250 °C [21,22]. The second stage, around 350 °C, is associated with the decomposition of the PEI core, as shown by Acebo et al [18] and by ourselves [19] in studies of the thermal degradation of PEI.

**Figure 3:**
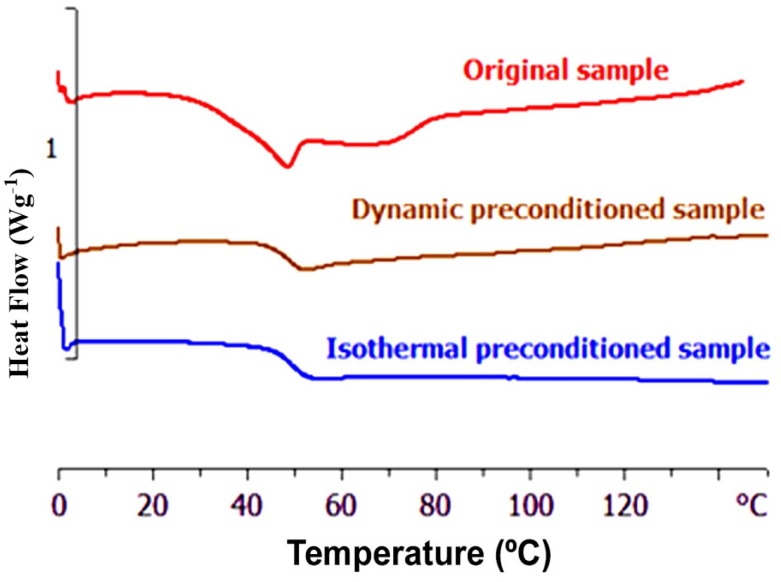
Differential scanning calorimetry (DSC) plots of heat flow (Wg^−1^) as a function of temperature (°C), at a heating rate of 10 °C/min, for PEI2000-PLA5 in the original and preconditioned states. The heat flow scale (*y*-axis) is relative, and the curves have been separated for clarity.

Figure 3 shows the thermogram obtained by differential scanning calorimetry (DSC, Mettler-Toledo AG), at the heating rate of 10 °C/min, for the PEI2000-PLA5 sample in its original state (upper curve). An endothermic peak at approximately 50 °C followed by a shoulder is observed. This peak has been attributed to the glass transition of PEI2000-PLA5 influenced by the physical aging effect and possibly by the presence of moisture (slight plasticization). In order to eliminate the effects of physical aging and humidity, the samples were subjected to two different preconditioning processes: dynamic and isothermal. For dynamic preconditioning, a non-isothermal scan was made at 10 °C/min over a temperature range from 0 to 150 °C, while for isothermal preconditioning, the sample was held at 100 °C for 30 min, each preconditioning procedure being followed by cooling to 0 °C before starting the DSC scan in which the sample is heated at a rate of 10 °C/min from 0 to 150 °C. For such preconditioned samples, Figure 3 shows thermograms (middle curve, dynamic preconditioning; lower curve isothermal preconditioning) in which only the glass transition of the material is observed. Table 2 shows the DSC results for all samples investigated. Both methods of preconditioning give the same glass transition temperature in all the samples studied. The preconditioned samples have a glass transition temperature between 3 and 5 °C higher than the non-preconditioned samples as a consequence of the removal of moisture.

The glass transition temperature, *T*_g_, of the three samples tested, with different molecular weights of the poly(ethyleneimine) core and different degrees of polymerisation of the lactide arms in the star polymer, are essentially the same: 45.0 ± 1.5 °C in the original samples and 49.0 ± 1.0 °C in the preconditioned samples. It is noted that the *T*_g_ of each of the multiarm star polymers is much higher than those observed for the hyperbranched polymer, PEI*x*, which forms the core of the star polymer, for which the *T*_g_ is −54 °C for PEI2000 and −58.3 °C for PEI800. The presence of the PLA arms increases the glass transition temperature with respect to the PEI core. It is also noteworthy that the glass transition temperatures obtained for the PEI*x*-PLA*y* systems here are slightly lower than those obtained for samples of PLA alone, around 57 °C [23,24,25,26,27,28], and closer to that of a racemic amorphous PLA polymer, for which the *T*_g_ is 47 °C [29].

During the heating above the glass transition, the calorimetric curves of Figure 3 do not display any exothermic peak, which would be characteristic of cold crystallization, nor any peak associated with melting. Most DSC thermograms were made over the temperature range from 0 to 150 °C, but additional scans were also made for the multiarm star polymers over a wider temperature range, from −65 to 180 °C, including a first scan, a quench, and a second scan. This was done to verify that there was no residual glass transition of the PEI, and to demonstrate that PEI2000-PLA5, and also the PEI2000-PLA20 and PEI800-PLA5 systems, are amorphous and non-crystallizable, in comparison with quenched samples of PLA, for which cold crystallization occurs in the range 102–122 °C and the endothermic peak due to melting at 148–175 °C [23,24,26,27].

### Dielectric Relaxation Spectroscopy (DRS)

In this section, we present and discuss the results of dielectric relaxation spectroscopy (DRS, TA Instruments, Inc., New Castle, DE, USA) on each of the three systems (PEI2000-PLA5, PEI2000-PLA20, PEI800-PLA5) separately, and then compare the behaviors in order to identify the effects of the different molecular structures (PEI core and PLA arms) and hence obtain information about the molecular relaxation processes.

#### (1) PEI2000-PLA5

**Figure 4:**
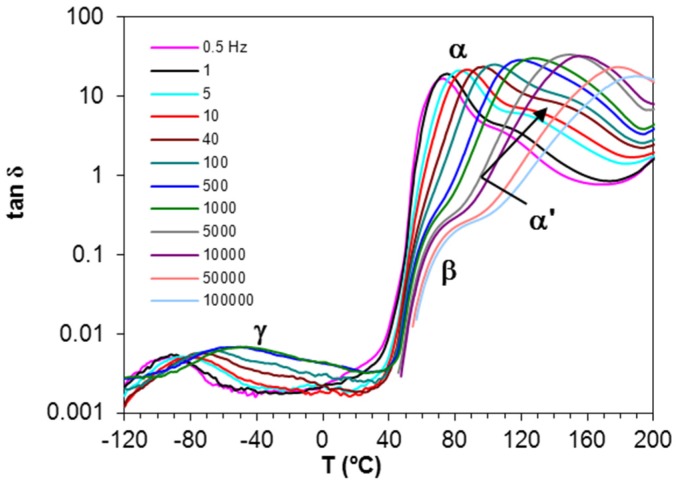
Dielectric relaxation spectroscopy curves (DRS) for PEI2000-PLA5 at 0.5 °C/min over the frequency range from 0.5 Hz to 100 kHz, as defined in the inset.

Dielectric relaxation curves for PEI2000-PLA5, PEI2000-PLA20, and PEI800-PLA5 preconditioned samples were obtained at 0.5 and 2 °C/min, over the frequency range from 0.1 Hz to 100 kHz, in order to obtain the dielectric constant ε′, loss factor ε′′, and tan δ signals. As an illustration, Figure 4 shows the dielectric relaxation curves for the PEI2000-PLA5 sample. 

**Figure 5:**
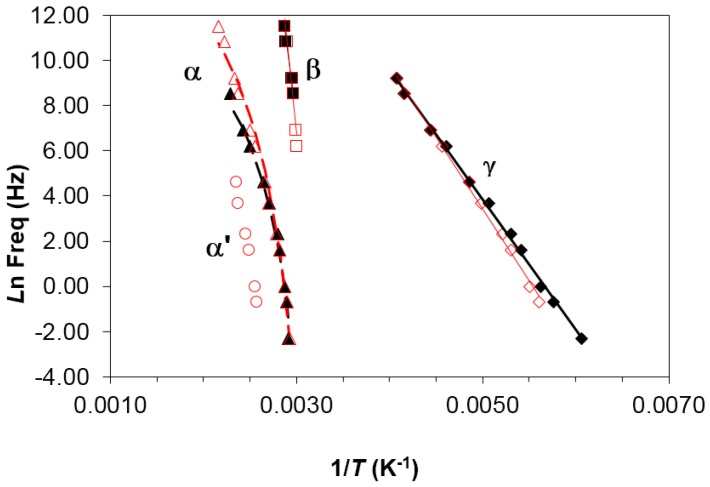
Relaxation map for PEI2000-PLA5 sample at different heating rates: 0.5 °C/min (open symbols) and 2 °C/min (filled symbols).

Four dipolar relaxations can be observed in the dielectric relaxation curves of PEI2000-PLA5: these are labelled γ, β, α, and α′, from lowest to highest temperature. The γ-relaxation appears between −90 and −50 °C depending on the measurement frequency, shifting to higher temperature as the frequency increases. The β-relaxation is hidden at low frequencies, but appears as a shoulder at frequencies above 500 Hz, in the temperature interval between 70 °C (0.5 kHz) and 90 °C (100 kHz), increasing in temperature as the frequency increases. In order to determine approximately the frequency dependence of this β-relaxation, an “onset” temperature is found from the intersection of tangents before and after the shoulder, and is used instead of a peak temperature. The α-relaxation is the dominant relaxation, the peak temperature increasing from around 80 °C at the frequency of 1 Hz to 190 °C at 100 kHz. A shoulder, associated with the relaxation labelled α', is observed at temperatures above the α-relaxation. The relaxation map for PEI2000-PLA5 at different heating rates is shown in Figure 5.

It can be seen that the heating rate has virtually no influence on the relaxation behavior. An Arrhenius dependence on temperature can be seen for the γ- and β-relaxations, from which the activation energy, averaged for the two heating rates used, was found as 50.2 ± 2.3 kJ/mol for the γ-relaxation and 267 ± 15 kJ/mol for the β-relaxation. The activation energy values obtained for each heating rate are shown in Table 3.

In contrast, the α-relaxation shows a slight curvature which is characteristic of the Vogel–Fulcher–Tammann (VFT) equation:

ln (*f*_m_) = *A* − *B*/(*T* − *T*_0_) (1)

where *f*_m_ is the frequency for which the maximum in tan δ occurs at the temperature *T*. The parameters *A*, *B,* and *T*_0_ of the VFT equation can be determined as follows. Selecting an initial value of *T*_0_ between *T*_g_ − 30 and *T*_g_ − 70, the values of *A* and *B* are obtained by a least squares fit of the experimental data, together with the degree of fit. This procedure is repeated for successive values of *T*_0_ until the best possible fit is obtained, which thus defines the required values of *T*_0_, *A,* and *B*. The values obtained for *A*, *B,* and *T*_0_ in this way for PEI2000-PLA5 are: *A* = 18.6 ± 2.0, *B* = 1583 ± 314 K, and *T*_0_ = 282.5 ± 7.5 K, being the average of the values obtained for the different heating rates. Given that *T*_g_ for this sample is 321.5 K by DSC (see Table 2), it can be seen that *T*_0_ is approximately 40 K less than the calorimetric *T*_g_. Table 3 lists the results obtained for each heating rate. As well as fitting the VFT equation, an “apparent” activation energy, *E*_app_, for the α-relaxation can be determined from the slope of a best fit straight line to the data, which gives values of 264 kJ/mol and 241 kJ/mol for the heating rates of 2 and 0.5 °C/min, respectively (average value 252 ± 12 kJ/mol).

#### (2) PEI2000

**Figure 6:**
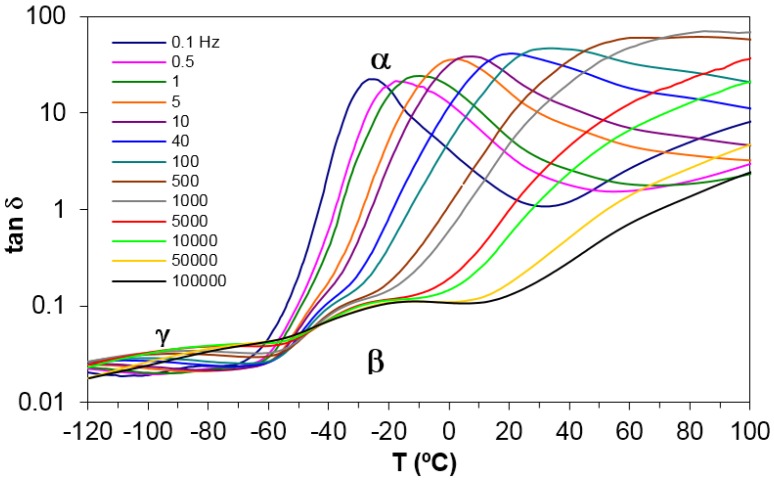
Dielectric relaxation spectroscopy curves for the PEI2000 sample at 0.5 °C/min, for the frequency range from 0.1 Hz to 100 kHz.

Figure 6 shows the relaxation curves for PEI2000, poly(ethyleneimine), obtained under the same conditions as for PEI2000-PLA5, in order to compare the relaxation peaks of both samples and hence elucidate their possible origins. In these spectra for PEI2000, the γ-relaxation can be seen at around −90 °C at the frequency of 1 Hz, and it moves to higher temperatures with increasing frequency. This relaxation peak is attributed to local motions of the –NH_2_ groups of PEI [19]. The relaxation map for different heating rates is depicted in Figure 6, where the linear relationship between ln(*f*_m_) and 1/*T* indicates an Arrhenius behavior, for which the activation energy associated with this process is found as 41.6 ± 0.4 kJ/mol (see Table 3), somewhat lower than but of the same order of magnitude as the activation energy for the γ-relaxation of PEI2000-PLA5 (50.2 ± 2.1 kJ/mol).

**Figure 7:**
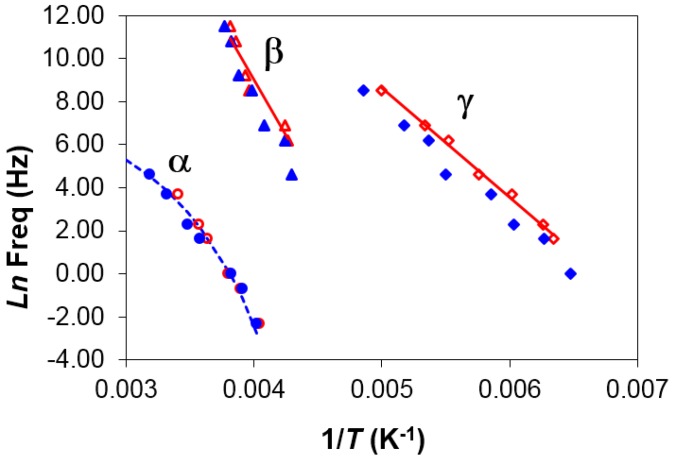
Relaxation map for PEI2000 at different heating rates: 0.5 °C/min (open symbols) and 2 °C/min (filled symbols).

The β-relaxation of PEI2000, a dipolar relaxation, is observed at temperatures above the γ peak, appearing first as a shoulder at a temperature of about −40 °C at a frequency of 0.1 kHz, and moving to higher temperatures and becoming more pronounced with increasing frequency. This relaxation is associated with motions of the lateral branches of PEI [19]. The average activation energy, calculated from the slope of the linear representation in Figure 7, is 92.7 ± 6.3 kJ/mol. In contrast to the comparison of the γ-relaxations, this activation energy for the β-relaxation of PEI2000 is much lower than the average value for the β-relaxation of PEI2000-PLA5 (267.0 ± 14.8 kJ/mol, Table 3). This indicates that the two relaxations have a different origin: the PLA arms in the PEI*x*-PLA*y* system do not exist in PEI2000. 

The α-relaxation, associated with the glass transition of PEI, is observed at temperatures higher than those of the β-relaxation, and the inverse of the peak temperatures obtained for the different frequencies are plotted in the relaxation map of Figure 7. In the same way as for the PEI*x*-PLA*y* systems, a non-linear relationship is observed, though it is less well defined as the frequency range is smaller. If this behavior is fitted by the VFT equation, we obtain the results shown in Table 3 for the different heating rates used. The average results for the different heating rates are *A* = 21.9 ± 3.2, *B* = 3709 ± 804 K, and *T*_0_ = 95.5 ± 12.8 K. As can be seen, the value obtained for the parameter *B* is very high and *T*_0_ is very low, more than 120 K below *T*_g_, which does not seem reasonable. This is possibly a consequence of an insufficient range of temperatures and frequencies to obtain reliable values for these parameters. Accordingly, the apparent activation energy, *E*_app_, from the average slope of a best fit straight line to the data for the α-relaxation provides a more reliable means of comparing the two systems. For PEI, *E*_app_ takes values of 78.1 kJ/mol and 63.3 kJ/mol for the heating rates of 0.5 and 2 °C/min, respectively, the average value being 70.7 ± 7.4 kJ/mol. This is noticeably less than that for PEI2000-PLA5, 252 ± 12 kJ/mol, suggesting a much higher degree of co-operativity in the molecular motions associated with the glass transition of the PEI*x*-PLA*y* systems.

The α-relaxation peak is an asymmetric peak in which the presence of a shoulder on the high temperature side can be clearly observed in Figure 6. The presence of this shoulder seems to suggest the existence of a relaxation peak, overlapping the dipolar α-relaxation and associated with ionic charge trapped in the PEI, which could be labelled as an α′-relaxation. This is supported by other results, not shown here but to be reported in another paper, where the effect of the molecular weight of the PEI core, up to 25000, on this ionic relaxation is revealed. It is observed there that, with increasing molecular weight of the PEI core, this shoulder corresponding to the ionic α′-relaxation becomes more pronounced and is clearly evident over a wider frequency range.

#### (3) PLA

Many dielectric studies of amorphous poly(lactide) can be found in the literature, which show that there are three dielectric relaxations, usually designated as α_n_, α_s,_ and β in order of decreasing temperature [30]. The α_n_-relaxation is assigned to the normal mode relaxation due to the component of dipole vector aligned in the direction parallel to the chain contour and the fluctuation of the end-to-end vector, and the α_s_-relaxation is assigned to the local segmental modes associated with the glass transition. The β-relaxation is observed at temperatures well below *T*_g_, and hence is attributed to secondary relaxations in the glassy state, and in particular to twisting motions of the main chains as there are no flexible side groups on which dipoles are attached [28,30].

Pluta et al [27] found that the temperature dependence of the α_s_-relaxation follows the VFT Equation (1) with *T*_0_ = 302 K and *B* = 800 K, which are stated to be in reasonable agreement with other results [29,30]. This value of *T*_0_, though, is only 28 K below the measured *T*_g_ of 330 K, which seems rather low. Accordingly, we converted these values into an apparent activation energy of 354 kJ/mol at an average temperature of 350 K, for comparison with our own results listed in Table 3. This is a very high value for the activation energy, and it is consistent with the observation that incorporating the PLA arms onto the PEI2000 core molecule results in a much higher activation energy for the PEI2000-PLA5 sample in comparison with that for the PEI2000 alone. Indeed, as is shown below, the activation energy for the glass transition increases significantly relative to the PEI core in all the PEI*x*-PLA*y* systems studied here.

Pluta et al [27] also reported a secondary β-relaxation in PLA, with an activation energy of 41.5 kJ/mol, in reasonable agreement with other values in the literature: 39.1 kJ/mol [28]; 43.9 kJ/mol [29]; 41.1 kJ/mol [30]; 36.4 kJ/mol [31].

The γ-relaxation for PEI2000-PLA5, whose maximum is located between –90 °C at 1 Hz and –50 °C at 1 kHz, corresponds approximately with the temperature of the β-relaxation in PLA (–80 °C) at a frequency of 1 Hz, though the average activation energy, 50.2 ± 2.3 kJ/mol for PEI2000-PLA5, is somewhat higher than that of the β-relaxation of PLA (36.4 to 43.9 kJ/mol, as listed above). Accordingly, the γ-relaxation in PEI2000-PLA5 could be associated with the terminal –OH groups of the PLA arms, with some additional hindrance resulting from the PLA being incorporated as the arms of the star-shaped polymer. The γ-relaxation of the PEI2000, associated with terminal –NH_2_ groups, is also observed in the same temperature range and its activation energy (41.6 ± 0.4 kJ/mol) is similar to that of PEI2000-PLA5. However, the γ-relaxation of PEI2000-PLA5 cannot be correlated with the γ-relaxation of PEI2000, because the movements of the –NH_2_ groups should not be seen, as a consequence of their replacement by PLA chains.

The β-relaxation of PEI2000-PLA5 is only observed above 0.5 kHz, and then only as a superimposed shoulder, at about 80 °C, on the low temperature flank of the α-relaxation. The activation energy for the β-relaxation of PEI2000-PLA5 is 267 ± 15 kJ/mol, which is much higher than the β-relaxation of PEI2000 (92.7 ± 6.3 kJ/mol). This suggests that this relaxation could be due to movements of the entire chain of PLA which forms the arms of the multiarm star polymer. The segmental motions associated with the glass transition of PLA have a VFT temperature dependence, from which one can estimate an apparent activation energy of about 114 kJ/mol at 350 K [24,30], approximately the same temperature as that at which the β-relaxation of PEI2000-PLA5 is observed. The implication of this would be that these segmental motions of the PLA arms are considerably restricted if they appear as the β-relaxation in the PEI*x*PLA*y* multiarm star polymers.

On the other hand, the α-relaxation of PEI2000-PLA5, occurring at 70 °C at 1 Hz, is associated with the glass transition of the polymer and could correspond to the α_s_-relaxation of the PLA (occurring at about 60 °C at 1 Hz [27]), but displaced to higher temperatures by the effect of the PEI2000 core. The α′-relaxation of PEI2000-PLA5 (90 °C at 1 Hz) corresponds to the α_N_-relaxation of the PLA (85 °C at 1 Hz [27]), influenced by the PEI core [27], without excluding the possibility that it could be a Maxwell–Wagner–Sillars type ionic peak because the material may have nano-regions of different conductivity. Above 1 kHz, the α- and α′-relaxations overlap and cause a unique dipolar relaxation. As seen in Figure 5, the relationship between ln(*f*_m_) and *T*^−1^ is practically linear, from which the apparent average activation energy for the α′-relaxation in the PEI2000-PLA5 is found as 263 ± 38 kJ/mol.

#### (4) PEI2000-PLA20

**Figure 8:**
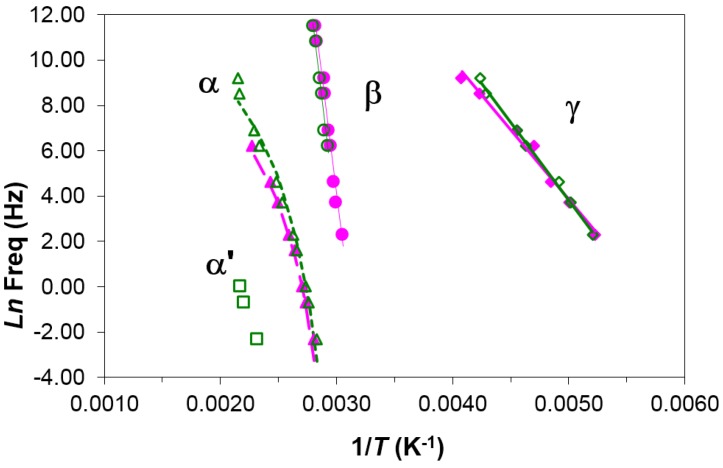
Relaxation map for PEI2000-PLA20 at different heating rates: 0.5 °C/min (open symbols) and 2 °C/min (filled symbols).

The multiarm star polymer PEI2000-PLA20 has the same structure as PEI2000-PLA5, but with the ratio of lactide repeat units to N links now being 20 instead of five. The dielectric relaxation curves for PEI2000-PLA20 are virtually identical to those for PEI2000-PLA5. The relaxation map, shown in Figure 8, indicates the existence of γ, β, α, and α′ relaxations, very similar to the relaxation map for PEI2000-PLA5 (Figure 5) but with some small displacements as a consequence of the effect of increasing the number of lactide repeat units.

**Figure 9:**
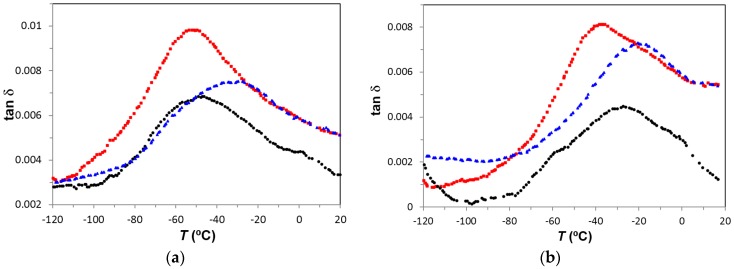
Dielectric relaxation spectroscopy curves for the γ-relaxation for the different samples studied: PEI2000-PLA5 (black circles), PEI2000-PLA20 (red squares) and PEI800-PLA5 (blue triangles) at frequencies: (**a**) 1 kHz; (**b**) 10 kHz. Heating rate: 0.5 °C/min.

The γ-relaxation (local motions of the –OH groups) is observed in PEI2000-PLA20 at temperatures lower than those for PEI2000-PLA5. Thus, at 1 kHz, γ-relaxation of PEI2000-PLA5 appears at −48 °C while that of PEI2000-PLA20 appears at −53 °C (−27 and −37 °C for 10 kHz, respectively), Figure 9a,b. The activation energy is similar in both polymers (50.2 ± 2.3 kJ/mol in PEI2000-PLA5 and 54.0 ± 3.2 kJ/mol in PEI2000-PLA 20). Increasing the ratio of lactide repeat units to N links, reduces the temperature at which the γ-relaxation occurs but does not significantly affect the activation energy, which is also relatively unaffected by the heating rate (57.2 kJ/mol at 0.5 °C/min and 50.8 kJ/mol at 2 °C/min for PEI2000-PLA20), Table 3. Figure 9a,b also shows that the intensity of the relaxation is greater in PEI2000-PLA20 than in PEI2000-PLA5, regardless of heating rate and frequency, this effect being related to the mobility of a larger number of OH groups present in the PEI2000-PLA20 structure.

**Figure 10:**
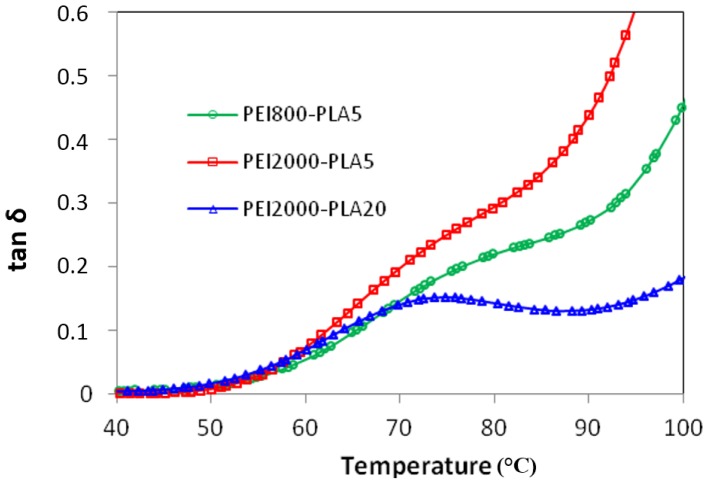
Dielectric relaxation spectroscopy curves for the β-relaxation for the different samples studied: PEI2000-PLA5, PEI2000-PLA20 and PEI800-PLA5. Frequency: 100 kHz. Heating rate: 0.5 °C/min.

The β-relaxation of the PEI2000-PLA20, due to movements of the entire chain of PLA, occurs as a shoulder at low frequencies but develops as a peak as the frequency increases. Figure 10 shows the β-relaxation of PEI2000-PLA5 compared with PEI2000-PLA20 at 100 kHz for the heating rate of 0.5 °C/min. For PEI2000-PLA5, a shoulder can be seen between 60 and 85 °C, while for PEI2000-PLA20 there is a clear peak with a maximum around 75 °C. This is because the higher ratio of lactide repeat units to N links in PEI2000-PLA20 results in a higher glass transition temperature, and hence a greater separation of the β- and α-relaxations. The relaxation map, Figure 8, shows that this β-relaxation is insensitive to the heating rate.

**Figure 11:**
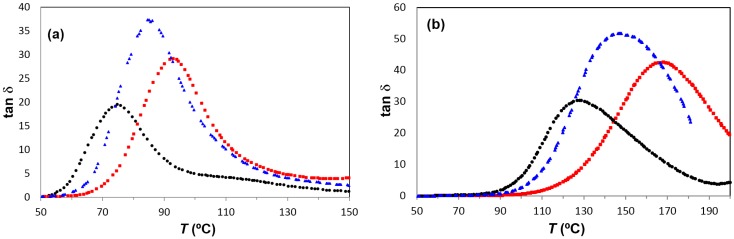
Dielectric relaxation spectroscopy curves for the α-relaxation for the different samples studied: PEI2000-PLA5 (black circles), PEI2000-PLA20 (red squares) and PEI800-PLA5 (blue triangles), at frequencies: (**a**) 1 Hz; (**b**) 1 kHz. Heating rate: 0.5 °C/min.

The α-relaxation is observed in PEI2000-PLA20 at higher temperatures than in PEI2000-PLA5, and this difference increases with increasing frequency. For example, as seen in Figure 11, the α-relaxation is observed at 75 °C in PEI2000-PLA5 and at 93 °C in PEI2000-PLA20 for the frequency of 1 Hz, while for 1 kHz these temperatures are 127 and 169 °C, respectively.

On the relaxation map in Figure 8, the representation of ln(*f*_m_) vs. *T*^−1^ for PEI2000-PLA20 is non-linear for the α-relaxation, adjusting to the VFT Equation (1). The parameters of this equation are given in Table 3 for the heating rates studied, the average values being: *A* = 15.1 ± 1.1, *B* = 1398 ± 173 K, and *T*_0_ = 274.5 ± 6.4 K. Comparing the results for PEI2000-PLA5 and PEI2000-PLA20, it can be seen that, for the same heating rate, the parameters of the VFT equation are lower in the PEI2000-PLA20. The apparent activation energy, calculated from the average slope of a best fit straight to the experimental data, is *E*_app_ = 170.0 ± 1.2 kJ/mol for the PEI2000-PLA20, and is lower for PEI2000-PLA20 than for PEI2000-PLA5, possibly as a consequence of the greater flexibility of the PLA20 chains.

**Figure 12:**
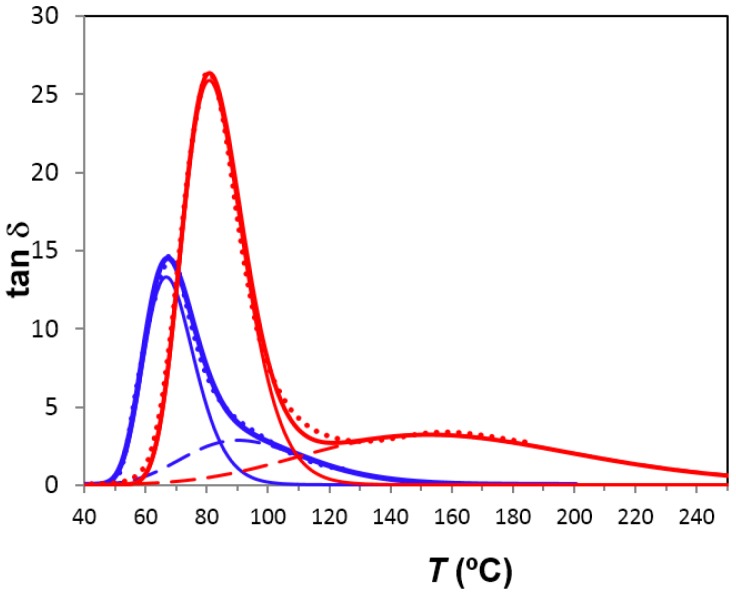
Deconvolution of the dielectric relaxation spectroscopy curves for PEI2000-PLA5 (blue), PEI2000-PLA20 (red): experimental curves (dotted lines); fit (thick lines), deconvoluted into α-relaxation (thin lines) and α′-relaxation (dashed lines). Frequency: 0.1 Hz. Heating rate: 0.5 °C/min.

The dipolar α′-relaxation appears at temperatures higher than the α-relaxation. This relaxation is more visible on decreasing both the frequency and the heating rate. In PEI2000-PLA20, this relaxation peak is better defined than in the PEI2000-PLA5 due to the higher ratio of lactide repeat units to N links, as shown in Figure 12. The relaxation map in Figure 8 shows that there is a linear relationship between ln(*f*_m_) and *T*^−1^ over the rather limited frequency range available, from which the activation energy is found as 131.0 kJ/mol, for the heating rate of 0.5 °C/min, significantly less than that obtained for PEI 2000-PLA5 at the same heating rate.

#### (5) PEI800-PLA5

**Figure 13:**
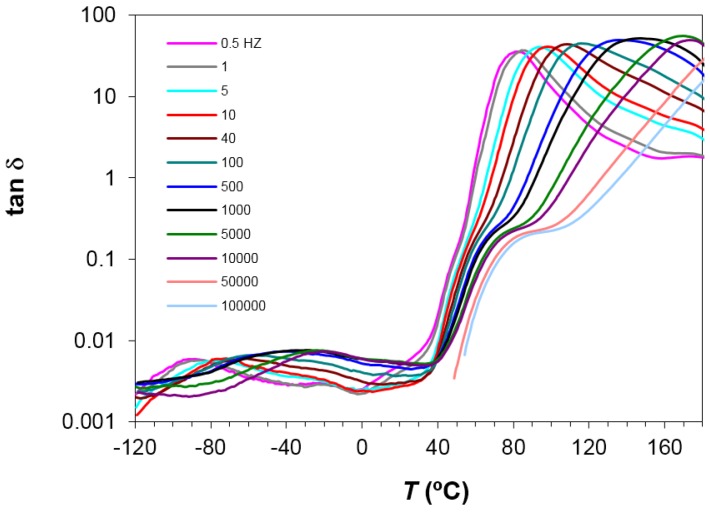
Dielectric relaxation spectroscopy curves for PEI800-PLA5 at 0.5 °C/min for frequencies between 0.5 Hz and 100 kHz.

The multiarm star polymer PEI800-PLA5 has the same ratio (= 5) of lactide repeat units to N links as for PEI2000-PLA5, but a lower molecular weight for the hyperbranched poly(ethyleneimine) core. The dielectric relaxation curves are similar to that for PEI2000-PLA5, and are shown in Figure 13.

**Figure 14:**
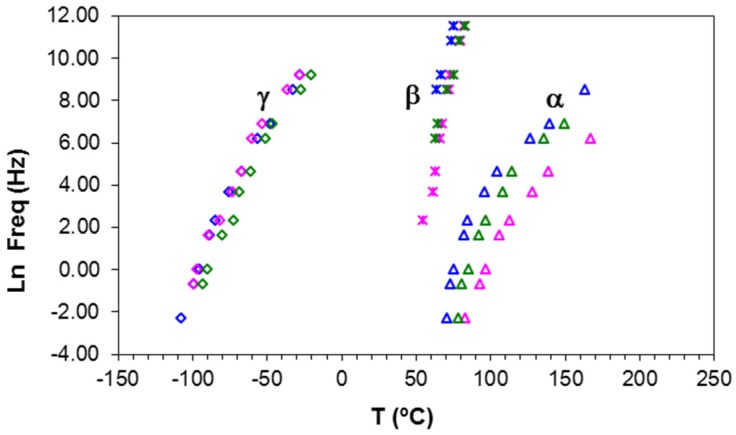
Relaxation map for PEI800-PLA5 (green), PEI2000-PLA5 (blue) and PEI2000-PLA20 (pink) at heating rate 2 °C/min: α-relaxation (triangles), β-relaxation (asterisks), γ-relaxation (rhombus).

**Figure 15:**
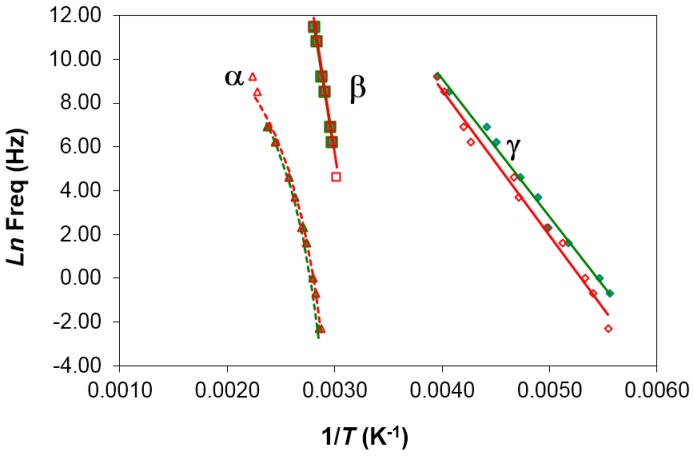
Relaxation map for PEI800-PLA5 at different heating rates: 2 °C/min (filled symbols) and 0.5 °C/min (open symbols).

The γ-relaxation for PEI800-PLA5, associated with local movements of the hydroxyl groups, appears at a slightly higher temperature than for PEI2000-PLA5 and PEI2000-PLA20, as shown in Figure 14: at 1 Hz and 2 °C/min, the γ-relaxation appears a −90 °C for PEI800-PLA5, at −95 °C for PEI2000-PLA5, and at −97 °C for PEI2000-PLA20. Figure 9 compares the γ-relaxation for all three samples at two selected frequencies: 1 kHz and 10 kHz. It is noted that the magnitude of tan δ for PEI800-PLA5 is intermediate between PEI2000-PLA20 and PEI2000-PLA5, the highest value being for PEI2000-PLA20. This might appear to suggest that the position of the γ-relaxation on the temperature scale is determined principally by the average molecular weight of the PEI core, whereas the intensity of the relaxation peak is dominated by the density of the PLA branches. However, these two effects are inter-related, since a higher molecular weight PEI core will imply a higher density of the arms, and so the individual contributions of each cannot be distinguished by these results. The relaxation map of Figure 15 shows that this relaxation is practically independent of the heating rate. The average activation energy for PEI800-PLA5 is 53.7±1.4 kJ/mol, very close to those for PEI 2000-PLA5 and PEI2000-PLA20, 50.2±1.3 kJ/mol and 54.2±3.2 kJ/mol, respectively.

As was also observed in the other samples, the β-relaxation becomes more visible at frequencies higher than 100 Hz for PEI800-PLA5, when it appears as a shoulder between 70 and 90 °C, as seen in Figure 13. Using the same procedure of determining an “onset” temperature for the β-relaxation as was used earlier for PEI2000-PLA5, the frequency of the β-relaxation can be estimated, and the relaxation map, Figure 15, shows an Arrhenius relationship between ln(*f*_m_) and *T*^−1^ with an average activation energy of 261 ± 4 kJ/mol, independent of the heating rate. This is almost identical to the value for PEI2000-PLA5 (267 ± 15 kJ/mol), but both are smaller than that for PEI2000-PLA20 (354 ± 10 kJ/mol), as can be seen in Table 3. The results indicate that the β-relaxation is influenced by the content of lactide repeat units/N link, not being sensitive to the effect of the molecular weight of the PEI core.

When comparing the results for the β-relaxation of PEI800-PLA5 with those for PEI2000-PLA5 and PEI2000-PLA20, we observe that the β-relaxation is located in the same temperature range for all samples, as shown in Figure 10, but occurs as a shoulder on samples with the same content of PLA, PEI2000-PLA5, and PEI800-PLA5, while it appears as a peak in the multiarm star polymer of higher PLA content, PEI2000-PLA20, for which the glass transition is better separated from the β-relaxation.

Figure 11a,b show the α-relaxation for PEI800-PLA5 for frequencies of 1 Hz and 1 kHz and a heating rate of 0.5 °C/min. For the frequency of 1 Hz, the α-relaxation peak is observed at 85 °C. This temperature is intermediate between the peak temperatures obtained for PEI2000-PLA5 and PEI2000-PLA20, 75°C and 93 °C, respectively. This behavior is observed for all the frequencies used, as shown in Figure 14, the difference being more marked as the frequency increases; however, for the minimum frequency of 0.1 Hz, the values of the temperature of the α-relaxation peak, associated with the glass transition of these multiarm star polymers, are substantially similar, as was observed in the DSC results (see Table 2). The VFT parameters obtained for the PEI800-PLA5 are *A* = 15.1 ± 0.2, *B* = 1107.1 ± 298 K, and *T*_0_ = 285.7 ± 11.4 K. These parameters are intermediate between those for PEI2000-PLA5 (*A* = 18.6 ± 2.0, *B* = 1583.0 ± 314 K, and *T*_0_ = 282.5 ± 7.6 K) and PEI2000-PLA20 (*A* = 15.0 ± 1.1, *B* = 1398.8 ± 173 K and *T*_0_ = 274.0 ± 6.9 K), as given in Table 3. The average apparent activation energy, *E*_app,_ for this relaxation in PEI800-PLA5 is 194 ± 8 kJ/mol, intermediate between those for PEI2000-PLA5 (252 ± 11 kJ/mol) and PEI2000-PLA20 (170 ± 2 kJ/mol) and higher than the PEI800 (68.7 ± 2.6 kJ mol^−1^), see Table 3. To explain the fact that these results for PEI800 PLA5 are intermediate between those for the other two multiarm star polymers, we consider the following.

It has been observed that the α-relaxation associated with the glass transition and the apparent activation energies for PEI800-PLA5 are much higher than for the PEI800 core, which can be explained by the predominant effect of the branches of PLA. The structure of the PEI800-PLA5 is much more complex than that of PEI800 and this translates into greater difficulty of molecular movement and consequently a higher activation energy. Comparing the multiarm star polymers with the same PLA content but with different molecular weights for the core, we observe that the temperature at which the α-relaxation peak appears and the activation energy for PEI800-PLA5 are higher than for PEI2000-PLA5; this can be attributed to the reduction in the molecular weight of the PEI core giving a structure which allows greater proximity and interaction between the branches of the PLA, and therefore the co-operative molecular mobility associated with the branches of PLA and the PEI core is more hindered, and as a consequence the α-relaxation is displaced towards higher temperatures. On the other hand, as was explained in the comparative analysis of PEI2000-PLA5 and PEI2000-PLA20, by increasing the ratio of lactide repeat units to N links, the possibilities for interaction between PLA chains increases, and the temperature of the α-relaxation peak for PEI2000-PLA20 is greater than for the other two multiarm star polymers, PEI800-PLA5 and PEI2000-PLA5.

The α′-relaxation, associated with the normal mode relaxation due to fluctuations of the end-to-end vector in the PLA arms, is more pronounced in PEI2000-PLA20, and appears as a well-defined peak, whereas this relaxation for the other two star-shaped polymers appears as a shoulder, as shown in Figure 12. The average activation energy for this α′-relaxation has been determined as 254 kJ/mol in PEI800-PLA5, virtually identical to that for PEI2000-PLA5 (263 kJ/mol), and considerably greater than that for PEI2000-PLA20 (131 kJ/mol). This indicates a null effect of the PEI core on this relaxation, as expected, while an increase in the ratio of lactide repeat units to N links causes a sharp decrease in the activation energy and a better definition of the relaxation peak. This possible assignment of this relaxation does not exclude that it could be a Maxwell–Wagner–Sillars type ionic peak because the material may have nano-regions of different conductivity.

**Figure 16:**
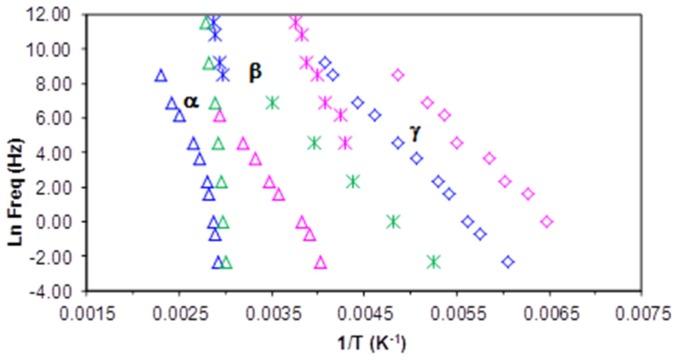
Relaxation map for PEI2000-PLA5 (blue), PEI2000 (pink), and PLA (green) at 2 °C/min: α-relaxation (triangles), β-relaxation (asterisks), γ-relaxation (rhombus).

Finally, we compare the multiarm star polymer PEI2000-PLA5 with PEI2000 and PLA on the relaxation map in Figure 16 in order to further clarify some of the ideas presented above. The data for amorphous PLA have been taken from Figure 5 of Laredo et al [32], with their secondary relaxation labelled β_III_ being taken as the β-relaxation. It is immediately apparent that the α-relaxation for PEI2000 is quite different from that for PEI2000-PLA5 whereas the relaxations for PEI2000-PLA5 and amorphous PLA merge at low frequency, and have approximately the same value of *T*_0,_ (as in fact do all the multiarm star polymers, see Table 3). The α-relaxation of all the multiarm star polymers is associated with the glass transition of the polylactide arms, with a displacement to higher temperatures as the frequency increases as a consequence of the restriction on mobility imposed by the PEI core. Likewise, the β-relaxation for PEI2000 can be seen to be widely separated from that of PEI2000-PLA5, and with a much lower activation energy (see Table 3), whereas again the relaxation in the multiarm star polymer appears to coincide with an extrapolation of the relaxation in the amorphous PLA. In fact, the β-relaxation in PEI2000-PLA5 closely superposes on the α-relaxation of the PLA in just the region in which the extrapolated β-relaxation of the PLA would intersect with the α-relaxation. This is consistent with the interpretation made in an earlier section that the β-relaxation in PEI2000-PLA5 was associated with the entire PLA chain, and supports the observation by Laredo et al [32] that the β-relaxation in PEI2000-PLA5 displays Johari-Goldstein characteristics. Lastly, Figure 16 also shows clearly the wide discrepancy between the γ-relaxations in PEI2000 and PEI2000-PLA5; even though they have similar slopes and hence similar activation energies (see Table 3), these relaxations are associated with quite different terminal groups.

## 3. Experimental

### 3.1. Materials

Multiarm star polymers were used, named generically PEI*x*-PLA*y*, where *x* represents the molecular weight of the poly(ethyleneimine) and *y* represents the ratio of lactide repeat units to N links. The three samples used were: PEI2000-PLA5, PEI2000-PLA20, and PEI800-PLA5, all in solid and powder form. The samples were provided by Acebo et al (Universitat Rovira i Virgili, Tarragona, Spain), who had previously made the synthesis and the characterization by ^1^H-NMR [18].

Polyethyleneimine (PEI) Lupasol^®^ FG (800 g/mol) and Lupasol^®^ PR 8515 (2000 g/mol) were donated by BASF. From the molecular weight of the polymer and of the repeating unit, average degrees of polymerization of 18.6 for Lupasol^®^ FG and 46.5 for Lupasol^®^ PR 8515 were calculated. The relationship (NH_2_/NH/N) was (1/0.82/0.53) for Lupasol^®^ FG and (1/0.92/0.70) for Lupasol^®^ PR 8515, and hence the equivalent number of primary, secondary and tertiary amines can be calculated as, respectively, 0.010, 0.00837, and 0.0053 eq/g for Lupasol^®^ FG and 0.0089, 0.0082, and 0.0062 eq/g for Lupasol^®^ PR 8515. Lactide (LA) was purchased from Sigma-Aldrich (Madrid, Spain). 

### 3.2. Experimental Techniques

Thermogravimetric analysis (TGA), which measures the weight loss of the sample during a programmed heating in a controlled atmosphere, was performed in a Mettler-Toledo TGA/DSC1 equipped with a sample robot and Huber cryostat (precision ± 0.1 °C). The TGA/DSC was calibrated using indium with a dry air flow of 200 mL/min. All the experiments were made with a dry nitrogen flow of 200 mL/min.

The DSC thermograms were studied using a Mettler-Toledo DSC 821e differential scanning calorimeter (DSC) equipped with a sample robot and Haake EK90/MT intracooler. All DSC experiments were performed with a dry nitrogen gas flow of 50 mL/min. The data evaluation was performed with the STARe software (Mettler-Toledo AG, Schwerzenbach, Switzerland). The DSC was calibrated for both heat flow and temperature using indium. A small sample of about 8–10 mg was weighed into an aluminium pan, sealed, and immediately inserted into the DSC furnace. The glass transition temperature, *T*_g_, was determined from the mid-point between the glassy and rubbery asymptotes. 

A dielectric analyzer DEA 2970 from TA Instruments was used to measure, in real time, the dielectric signals at different frequencies. Dielectric measurements were performed using a ceramic single-surface cell of 20 mm × 25 mm based on a coplanar inter-digitated comb-like electrode design. The values of dielectric permittivity, ε′, dielectric loss factor, ε′′, and tan δ (= ε′′/ε′) were obtained from the resulting current. The interval of data sampling was 1 s per point. The sample was spread on the electrode surface, covering the entire inter-digitated area. The non-isothermal measurements were performed at 2 and 0.5 °C/min in a nitrogen atmosphere with a gas flow of 500 mL/min. The ε′, ε′′, and tan δ were measured in the frequency range from 0.1 Hz to 100 kHz. In order to eliminate previous thermal history and any trace of moisture, the samples were preconditioned in the same dielectric analyzer as follows. The samples were heated from 25 °C to 100 °C at a rate of 2 °C/min, held isothermally at 100 °C for 30 min, and then cooled to −130 °C before scanning from−130 to 180 °C at the heating rates of 0.5 and 2 °C/min.

## 4. Conclusions

We studied three multiarm star PEI*x*-PLA*y* polymers with different molecular weights for the poly(ethyleneimine) (PEI) core (PEI*x*) and different ratios of lactide repeat units to N links (PLA*y*): PEI800-PLA5, PEI2000-PLA5, and PEI2000-PLA20. All samples were preconditioned for 30 min at 100 °C. The glass transition temperature of all samples, determined by DSC, was in the range 48–50 °C, independent of the molecular weight of the PEI core and of the ratio of lactide repeat units to N links. The glass transition temperatures of the multiarm star polymers were much higher than those for the PEI from which the multiarm star polymers were obtained.

The dielectric relaxation curves of all samples, for frequencies from 0.1 Hz to 100 kHz, show four dipolar relaxations: γ, β, α, and α′, in order of increasing temperature. The γ-relaxation for PEI800-PLA5 appears at a slightly higher temperature than for PEI2000-PLA5 and PEI2000-PLA20, and its peak intensity is intermediate between these other two samples. This suggests that the γ-relaxation can be influenced by both the molecular weight of the PEI core and the density of the multiarms, which are inter-related aspects of the molecular architecture. The relaxation map shows that this relaxation is independent of the heating rate, and has an Arrhenius temperature dependence, for which the average activation energy is 53.7 ± 1.4 kJ/mol, practically identical to those found for PEI2000-PLA5 and PEI2000-PLA20, 50.2 ± 2.3 kJ/min and 54.0 ± 3.2 kJ/min, respectively. The γ-relaxation is attributed to local motions of the terminal –OH groups of the poly(lactide) arms.

The β-relaxation becomes visible between 70 and 90 °C at frequencies higher than 100 Hz for PEI800-PLA5, but appears only as a shoulder on the α-relaxation peak. The combined effect of higher molecular weight PEI core (PEI*x*) and the highest ratio of lactide repeat units to N links (PLA*y*) results in the appearance of a clearly observable peak for PEI2000-PLA20. From the analysis of the relaxation map for the β-relaxation, we conclude that this relaxation is influenced by the content of lactide repeat units/N link and is not sensitive to the effect of the molecular weight of the PEI core. In particular, the activation energy for PEI800-PLA5 is approximately equal to that for PEI2000-PLA5, but significantly lower than that for PEI2000-PLA20. The β-relaxation is attributed to motions of the main chain of the poly(lactide) arms.

The dipolar α-relaxation, of the VFT type, appears at temperatures which depend on both the molecular weight of the PEI core and the ratio of lactide repeat units to N links. Independently of frequency, the PEI core and the PLA arms have opposing effects: increasing the molecular weight of the PEI core results in a decrease in the α-relaxation temperature (*T*_α_ PEI2000-PLA5 < *T*_α_ PEI800-PLA5), whereas increasing the ratio of lactide repeat units to N links results in an increase in the α-relaxation temperature (*T*_α_ PEI2000-PLA5 < *T*_α_ PEI2000-PLA20), the effect of the PLA arms being dominant (*T*_α_ PEI800-PLA5 < *T*_α_ PEI2000-PLA20). The apparent activation energy, *E*_app_, for this relaxation increases with decreasing ratio of lactide repeat units to N links while it is little influenced by the molecular weight of the PEI core (170 ± 1.2 kJ/mol PEI2000-PLA20 < 194.3 ± 7.5 kJ/mol PEI800-PLA5 < 252.4 ± 11.3 kJ/mol PEI2000-PLA5). The α-relaxation is attributed to global molecular motions of the assembly of PEI core and PLA arms.

The α′-relaxation, considered here to be associated with the normal mode relaxation due to fluctuations of the end-to-end vector in the PLA arms but recognizing also that it could be a Maxwell–Wagner–Sillars type ionic peak, is more pronounced in PEI2000-PLA20, for which it appears as a well-defined peak, in comparison with the other two multiarm star polymers for which it appears only as a shoulder, approximately within the same temperature interval. This indicates a null effect of the PEI core over this relaxation, as expected, while an increase in the ratio of lactide repeat units to N links causes a sharp decrease in the activation energy and a better definition of the relaxation peak.

## Figures and Tables

**Table 1:** Thermogravimetric analysis (TGA) results for the original and isothermally preconditioned samples.

Sample	Heating Rate (°C/min)	Original Sample		Preconditioned Sample	
		*T*_2%_ (°C)	*T*_onset_ (°C)	*T*_2%_ (°C)	*T*_onset_ (°C)
PEI2000-PLA5	10	93.3	234.5	176.4	243.9
PEI2000-PLA20	10	114.4	243.1	166.7	240.8
PEI800-PLA5	10	114.5	239.8	195.1	254.3

**Table 2:** Differential scanning calorimetry (DSC) results for the different samples investigated.

Sample	Heating Rate (°C/min)	*T*_g_ (°C)		
		Original Sample	Dynamic Preconditioned	Isothermal Preconditioned
PEI2000-PLA5	10	43.8	48.2	48.8
PEI2000-PLA20	10	45.7	48.6	48.8
PEI800-PLA5	10	46.5	49.1	50.4
PEI 800	10	−58.3	-	-
PEI 2000	10	−54.0	-	-

**Table 3:** Activation energy values *E*_a_ and Vogel–Fulcher–Tammann (VFT) parameters for the different samples studied.

Sample	Heating Rate (°C/min)	γ Relaxation	β Relaxation	α Relaxation			
				*E*_app_ (kJ/mol)	VFT Parameters		
		*E*_a_ (kJ/mol)	*E*_a_ (kJ/mol)		*A*	*B* (K)	*T*_0_ (K)
PEI2000-PLA5	0.5	52.5	282	241	20.3	1898	291
	2	48.0	252	264	16.3	1268	275
PEI2000-PLA20	0.5	57.2	364	169	16.2	1572	268
	2	50.8	345	171	13.9	1226	281
PEI800-PLA5	0.5	55.1	265	187	16.8	1405	274
	2	52.3	258	202	13.4	809	297
PEI2000	0.5	42.0	86.4	78.1	25.1	4512	82
	2	41.2	99.0	63.3	18.7	2905	109
PEI800	0.5	46.2	181	71.3	22.4	3544	88
	2	45.8	155	66.6	15.5	1459	156
PLA [28]	-	-	39.1	-	35.9	1840	279
PLA [30]	-	-	41.1	-	-	-	-
